# Association of *ALOX5AP* gene single nucleotide polymorphisms and cerebral infarction in the Han population of northern China

**DOI:** 10.1186/1471-2350-13-61

**Published:** 2012-07-31

**Authors:** Shuang-yan Zhang, Mei-ling Xu, Cui-e Zhang, Zheng-yi Qu, Bin-bin Zhang, Zu-yan Zheng, Li-ming Zhang

**Affiliations:** 1 Department of Neurology, The Fourth Affiliated Clinical College, Harbin Medical University, Yiyuan Street 37, Nangang District, Harbin, China; 2 Department of Neurology, The First Affiliated Hospital Heilongjiang, University of Chinese Medicine, Heping Road 26, Xiangfang District, Harbin, China; 3 Department of Neurology, The First Affiliated Clinical College, Harbin Medical University, Youzheng Street 23, Nangang District, Harbin, China

**Keywords:** Cerebral infarction, ALOX5AP, FLAP, Han ethnicity, SNP

## Abstract

**Background:**

To explore the association of *ALOX5AP* single nucleotide polymorphisms (SNPs) and haplotype with the occurrence of cerebral infarction in the Han population of northern China.

**Methods:**

Blood samples were collected from 236 patients of Han ancestry with a history of cerebral infarction and 219 healthy subjects of Han ancestry with no history of cerebral infarction or cardiovascular disease. Applied Biosystems® TaqMan® SNP Genotyping Assays for SNP genotyping were used to determine the genotypes of 7 *ALOX5AP* SNP alleles (rs4073259, rs4769874, rs9315050, rs9551963, rs10507391, rs9579646, and rs4147064).

**Results:**

One SNP allele (A) of rs4073259 was significantly associated with development of cerebral infarction (*P* = 0.049). In comparison to control groups, haplotype rs9315050&rs9551963 AAAC [OR (95% CI) =1.53 (1.02-2.29)], and genotypes rs4147064 CT [OR (95% CI) =1.872 (1.082-3.241)], and rs9551963 AC [OR (95% CI) = 2.015 (1.165-3.484)] increased the risk of cerebral infarction in patients with hypertension. Genotype rs9579646 GG [OR (95% CI) = 2.926 (1.18-7.251)] increased the risk of, while rs4073259 GG [OR (95% CI) = 0.381 (0.157-0.922)] decreased the risk of cerebral infarction in patients with diabetes.

**Conclusion:**

These results suggest the *ALOX5AP* SNP A allele in rs4073259 and genotype rs9579646 GG, rs9551963 AC, and haplotype rs9315050 & rs9551963 AAAC were associated with an increased risk of ischemic stroke in the Han population, while rs4073259 GG was associated with a decreased risk.

## Background

Multiple studies have shown associations between variants of the 5-lipoxygenase activating protein gene (*ALOX5AP*) and risk for ischemic stroke and myocardial infarction (MI) [1-9]. Two at-risk haplotypes (HapA and HapB) for stroke had been identified in the *ALOX5AP* gene [1,10]. The single nucleotide polymorphisms (SNPs) associated with HapA are rs17222814, rs10507391, rs4769874, and rs9551963, and those associated with the HapB are rs17216473, rs10507391, rs9315050, and rs17222842. Different studies, however, reported conflicting results. The association of a 4-marker SNP haplotype in *ALOX5AP* with approximately 2-times increased risk of ischemic stroke and myocardial infarction was reported in an Icelandic population [1]. A similar risk of stroke was found in Chinese males [5], Japanese [8], and Europeans [2]. On the contrary, a study found no association of *ALOX5AP* variants with risk of MI or stroke in the United States (US) by Zee *et al.*[6], whereas another study in the US by Kaushal *et al.*[9] found a significant association with stroke among whites, but no association among blacks. These reports suggest that there might be variations between the populations of different ancestries in the association between *ALOX5AP* genetic variants and risk of stroke.

The purpose of this study was to study the possible association of 7 variants of *ALOX5AP*, rs4073259, rs4769874, rs9315050, rs9551963, rs10507391, rs9579646, and rs4147064 with ischemic stroke in the population with Han ancestry in northern China.

## Material and methods

### Subjects

The subjects included in this study were patients diagnosed with cerebral infarction from three hospitals including the Second Affiliated Hospital of Harbin Medical University, the Forth Affiliated Clinical Hospital of Harbin Medical University, and the First Affiliated Hospital of Harbin Medical University from October 2004 to May 2010. All cases were diagnosed using the Fourth edition of Cerebrovascular Disease Diagnostic Standards [11]. Patients who had endocrine related diseases, metabolic disorders, tumors, and cerebral hemorrhage were excluded from this study. The control group was composed of subjects who did not have cerebrovascular diseases themselves, and no family history of cerebrovascular diseases for a minimum of three generations. All the subjects in this study were from different families of Han ethnicity whose families have lived in the Northeastern part of China, including Heilongjiang, Jilin and Liaoning, for more than 3 generations. This study was approved by the Institutional Review Board of the Fourth Affiliated Clinical Hospital of Harbin Medical University and each participant provided written informed consent.

### DNA extraction and genotyping

DNA was extracted in a standard manner. Applied Biosystems® TaqMan® SNP Genotyping Assays were used for SNP genotyping following the manufacturer’s instructions. In brief, one to 20 ng template DNA dissolved in 2.25 μL volume each well was loaded into 384-well plates for PCR. The reaction volume a total of 5 μL included 2.5 μL TaqMan Universal PCR master Mix (2x) and 0.25 μL 10× working stock of SNP genotyping assay buffer. The PCR reaction conditions were 95 °C for 10 min followed by 60 cycles of 92 °C for 15 sec and 60 °C for 1 min in an ABI 9700 PCR instrument. The genotyping data were collected by the ABI 9700 instrument. During SNP genotyping, duplicates/positive/negative controls were used as previously described [12].

Of the 4 HapA SNPs (rs17222814, rs10507391, rs4769874, and rs9551963), rs17222814 was not selected because in our preliminary study this SNP was shown to be almost monomorphic in the Chinese population. Thus, we selected 3 SNPs rs10507391, rs4769874, and rs9551963 from HapA for this study. Of the 4 HapB SNPs (rs17216473, rs10507391, rs9315050, and rs17222842), rs17222842 was excluded because it is monomorphic in the Chinese population, and rs17216473 was not selected because no significant association with stroke was noted in a preliminary study. The SNPs rs9579646 and rs4147064 were selected because they showed weak association with stroke in Israeli [2] and middle European populations [13], and rs4073259 was found to be associated with stroke in a preliminary study [12].

### Statistical analysis

Data are presented as mean ± standard deviation or number (percentage). Hardy-Weinberg equilibrium (HWE) testing was carried out for all 7 SNPs. Single markers in association with the disease were assessed using *χ*2 tests in Haploview Version 4.2 (Broad Institute, Cambridge, MA, USA). Odds ratios (ORs) and 95% confidence intervals (CIs) were calculated using logistic regression analysis. Statistical analysis was performed using by SPSS version 15.0 statistical software (SPSS, Inc., Chicago, IL, USA). A value of *P* < 0.05 was considered statistically significant.

## Results

### Patient demographics and disease characteristics

The characteristics of ischemic stroke patients and controls are summarized in Table 1. Patients with ischemic stroke were older and had higher triglyceride level than the controls (both *P* < 0.001). About 16.5% (n = 39), 37.3% (n = 88), and 22.9% (n = 54) of patients with ischemic stroke had also heart disease, hypertension or diabetes, respectively, while no control had these diseases (All *P* < 0.001). Cholesterol levels and gender distribution were not different between the groups.

**Table 1:** Patient demographics and disease characteristics

	**Ischemic Stroke Subjects (*n* = 236)**	**Healthy Control Subjects (*n* = 219)**	***P***
Age (y)	62.86 ± 11.34	50.36 ± 10.21	<0.001*
Triglycerides	1.75 ± 1.42	1.38 ± 0.93	<0.001*
Cholesterol	5.07 ± 1.16	5.00 ± 1.01	0.503
Male	125 (57.1)	145 (61.4)	0.390
Heart disease	39 (16.5)	0	<0.001*
Hypertension	88 (37.3)	0	<0.001*
Diabetes	54 (22.9)	0	<0.001*

Data are presented as mean ± standard deviation or number (percentage).

rs9579646/SG13S106 G 211 (48) 258(55) 0.0504

### Univariate analysis of SNP distribution between ischemic stroke patients and controls

The allele distribution of 7 SNPs of *ALOX5AP* was summarized in Table 2. Only the frequency of rs4073259 SNP allele A was significantly different between ischemic stroke patients and the controls (48% vs 54%, *P* = 0.049). The frequency of rs9579646 allele G had a marginal p value between the two groups (48% vs 55%, *P* = 0.0504).

**Table 2:** **The association of *ALOX5AP *SNP allele with ischemic stroke in northern Han Chinese **

**#**	**Public Name/SNP Name**	**Allele**	**Control Group (*n* = 219)**	**Case Group (*n* = 236)**	***P***
1	rs4147064/SG13S137	T	148 (34)	186 (39)	0.0882
		C	288 (66)	286 (61)	
2	rs9579646/SG13S106	G	211 (48)	258 (55)	0.0504
		A	227 (52)	214 (45)	
3	rs4073259/SG13S100	A	208 (48)	255 (54)	0.0487
		G	230 (53)	217 (46)	
4	rs10507391/SG13S114	T	269 (62)	304 (64)	0.3979
		A	167 (38)	168 (36)	
5	rs4769874/SG13S89	G	429 (98)	464 (98)	0.6887
		A	9 (2)	8 (2)	
6	rs9315050/SG13S41	A	429 (98)	464 (98)	0.6887
		G	9 (2)	8 (2)	
7	rs9551963/SG13S32	C	147 (34)	188 (4)	0.0564
		A	289 (66)	284 (60)	

Data are presented as numbers (percentages).

Then, the genotype distribution of ALOX5AP SNP in the ischemic stroke and the control groups is calculated (Table 3). Individuals with a genotype of rs9551963 AC or a genotype of rs9579646 GG had higher risks of developing a cerebral infarction than individuals with genotype rs9551963 AA or genotype rs9579646 AA (OR = 1.50 (1.01-2.24), *P* = 0.046, OR = 1.73 (1.01-2.97), *P* = 0.047, respectively), But individuals with a genotype of GG in the rs4073259 SNP had a lower risk (0.57-fold) of developing a cerebral infarction than individuals with the AA genotype (95% CI 0.33-0.98, P = 0.044).

**Table 3:** Genotype distribution in the stroke and control groups

**#**	**Public Name**	**Genotype**	**Control Group (*n* = 219)**	**Case Group (*n* = 236)**	**OR (95% CI)**	***P***
1	rs4147064	CC	97 (44)	85 (36)	Ref	--
		CT	94 (43)	116 (49)	1.41 (0.95-2.10)	0.092
		TT	27 (12)	35 (15)	1.48 (0.83-2.64)	0.186
2	rs9579646	AA	55 (25)	46 (19)	Ref	--
		AG	117 (53)	122 (52)	1.26 (0.79-2.01)	0.336
		GG	47 (21)	68 (29)	1.73 (1.01-2.97)	0.047*
3	rs4073259	AA	45 (21)	66 (28)	Ref	--
		AG	118 (54)	123 (52)	0.72 (0.45-1.13)	0.152
		GG	56 (26)	47 (20)	0.57 (0.33-0.98)	0.044*
4	rs10507391	AA	31 (14)	28 (12)	Ref	--
		AT	105 (48)	112 (47)	1.18 (0.66-2.10)	0.571
		TT	82 (38)	96 (41)	1.30 (0.72-2.34)	0.389
5	rs4769874	AG	9 (4)	8 (3)	Ref	--
		GG	210 (96)	228 (97)	1.09 (0.40-2.95)	0.872
6	rs9315050	AA	210 (96)	228 (97)	Ref	--
		AG	9 (4)	8 (3)	0.92 (0.34-2.50)	0.872
7	rs9551963	AA	99 (45)	84 (36)	Ref	--
		AC	91 (42)	116 (49)	1.50 (1.01-2.24)	0.046*
		CC	28 (13)	36 (15)	1.52 (0.85-2.69)	0.155

Data are presented as numbers (percentages) or odds ratio (OR) with 95% confidence interval (CI).

*Significant difference between control and case groups (*P* < 0.05).

For further analysis, a comparison of genotype distribution between the control group and case subgroups stratified into heart disease (n = 39), hypertension (n = 88), and diabetes (n = 54) is shown in Table 4. In comparison to controls, rs4147064 genotype CT [OR (95%CI) =1.872 (1.082-3.241)] and rs9551963 genotype AC [OR (95% CI = 2.015 (1.165-3.484)] were positively significantly associated with cerebral infarction in patients with hypertension. Genotype rs9579646 GG [OR (95% CI) = 2.926 (1.18-7.251)] was positively significantly associated with, while genotype rs4073259 GG [OR (95% CI) = 0.381 (0.157-0.922)] was negatively significantly associated with cerebral infarction in patients with diabetes.

**Table 4:** Comparison of genotype distribution between control and case groups stratified into heart disease, hypertension, and diabetes

**#**	**Public Name**	**Genotype**	**Control (*n* = 219)**	**Heart disease (*n* = 39)**		**Hypertension (*n* = 88)**		**Diabetes (*n* = 54)**	
				**OR (95% CI)**	***P***	**OR (95% CI)**	***P***	**OR (95% CI)**	***P***
1	rs4147064	CC	97 (44%)	1		1		1	
		CT	94 (43%)	1.096 (0.523-2.296)	0.8072	1.872 (1.082-3.241)	0.0251*	1.548 (0.8-2.995)	0.195
		TT	27 (12%)	1.347 (0.481-3.776)	0.5707	1.596 (0.715-3.562)	0.2534	1.796 (0.725-4.448)	0.2055
2	rs9579646	AA	55 (25%)	1		1		1	
		AG	117 (53%)	1.067 (0.437-2.604)	0.8871	1.171 (0.612-2.24)	0.6326	1.541 (0.655-3.623)	0.3215
		GG	47 (21%)	1.902 (0.726-4.981)	0.1908	1.996 (0.977-4.077)	0.0578	2.926 (1.18-7.251)	0.0204*
3	rs4073259	AA	45 (21%)	1		1		1	
		AG	118 (54%)	0.533 (0.241-1.176)	0.1189	0.591 (0.328-1.062)	0.0787	0.526 (0.266-1.043)	0.066
		GG	56 (26%)	0.495 (0.189-1.297)	0.1523	0.488 (0.238-1.001)	0.0504	0.381 (0.157-0.922)	0.0324*
4	rs10507391	AA	31 (14%)	1		1		1	
		AT	105 (48%)	1.475 (0.469-4.64)	0.5058	0.84 (0.398-1.776)	0.6485	1.771 (0.571-5.493)	0.3221
		TT	82 (38%)	1.417 (0.436-4.601)	0.5619	1.105 (0.52-2.347)	0.7949	2.457 (0.793-7.614)	0.1192
5	rs4769874	AG	9 (4%)	1		1		1	
		GG	210 (96%)	0.704 (0.144-3.448)	0.6655	1.079 (0.28-4.166)	0.9118	0.647 (0.166-2.526)	0.5312
6	rs9315050	AA	210 (96%)	1		1		1	
		AG	9 (4%)	1.42 (0.29-6.949)	0.6655	0.926 (0.24-3.576)	0.9118	1.545 (0.396-6.029)	0.5312
7	rs9551963	AA	99 (45%)	1		1		1	
		AC	91 (42%)	1.156 (0.552-2.422)	0.701	2.015 (1.165-3.484)	0.0122*	1.792 (0.92-3.489)	0.0863
		CC	28 (13%)	1.326 (0.474-3.706)	0.5906	1.44 (0.636-3.261)	0.3813	1.872 (0.753-4.652)	0.1771

Data are presented as numbers (percentages) or odds ratios (OR) with 95% confidence interval (CI).

*Significant differences between controls and case subgroups (*P* < 0.05).

### Analysis of haplotype distribution between ischemic stroke patients and controls

The haplotype distribution in the stroke and control groups are shown in Table 5. Patients with haplotype rs9315050&rs9551963 AAAC had a higher risk to have ischemic stroke than haplotype rs9315050&rs9551963 AAAA (OR = 1.53 (1.02-2.29), *P* = 0.041).

**Table 5:** Haplotype distribution in the stroke and control groups

**Public Name**		**Haplotype**	**Control Groups (*n* = 219)**	**Case Groups (*n* = 236)**	**OR (95% CI)**	***P***
rs4147064 & rs9579646		CCAA	36 (17%)	26 (11%)	Ref	--
		CCAG	50 (23%)	40 (17%)	1.11 (0.58-2.13)	0.759
		CCGG	11 (5%)	19 (8%)	2.39 (0.98-5.87)	0.057
		CTAA	15 (7%)	20 (8%)	1.85 (0.80-4.27)	0.152
		CTAG	53 (24%)	64 (27%)	1.67 (0.90-3.12)	0.105
		CTGG	26 (12%)	32 (14%)	1.70 (0.83-3.51)	0.148
		TTAA	4 (2%)	0 (0%)	--	--
		TTAG	13 (6%)	18 (8%)	1.92 (0.80-4.59)	0.144
		TTGG	10 (5%)	17 (7%)	2.35 (0.93-5.97)	0.071
rs4073259 & rs10507391		AAAT	6 (3%)	2 (1%)	Ref	--
		AATT	39 (18%)	64 (27%)	4.92 (0.95-25.61)	0.058
		AGAA	2 (1%)	3 (1%)	4.50 (0.41-49.63)	0.219
		AGAT	75 (34%)	90 (38%)	3.60 (0.71-18.36)	0.123
		AGTT	40 (18%)	30 (13%)	2.25 (0.42-11.94)	0.341
		GGAA	29 (13%)	25 (11%)	2.59 (0.48-13.98)	0.27
		GGAT	24 (11%)	20 (8%)	2.50 (0.45-13.78)	0.293
		GGTT	3 (1%)	2 (1%)	2.00 (0.18-22.06)	0.571
rs9315050 & rs9551963		AAAA	99 (45%)	84 (36%)	Ref	--
		AAAC	85 (39%)	110 (47%)	1.53 (1.02-2.29)	0.041*
		AACC	26 (12%)	34 (14%)	1.54 (0.86-2.77)	0.149
		AGAC	6 (3)%	6 (3%)	1.18 (0.37-3.79)	0.783
		AGCC	2 (1%)	2 (1%)	1.18 (0.16-8.55)	0.871

Data are presented as numbers (percentages) or odds ratios (OR) with 95% confidence interval (CI).

*Significant differences between control and case groups (*P* < 0.05).

**Figure 1:**
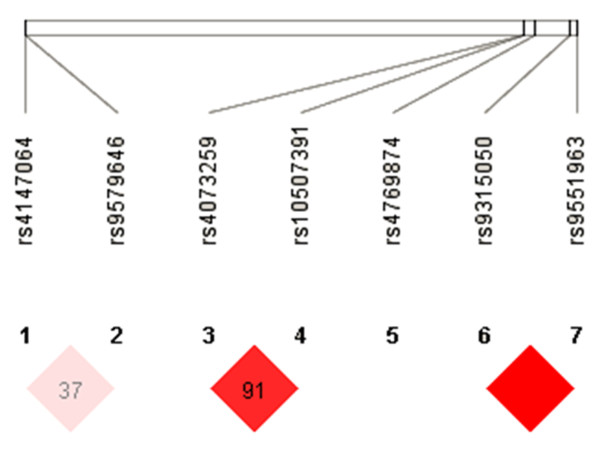
**Analysis of haplotype distribution between ischemic stroke patients and controls.** Linkage was found to be significant between rs9315050 *vs.* rs9551963, rs4073259 *vs.* rs10507391, and rs9579646 *vs.* rs4147064 (D’ = 1.0, D’ = 0.911, and D’ = 0.376, respectively).

We further did linkage disequllibrium analysis and found significant linkage between rs9315050 *vs.* rs9551963, rs4073259 *vs.* rs10507391, and rs9579646 *vs.* rs4147064 (D’ = 1.0, D’ = 0.911, and D’ = 0.376, respectively, Figure 1).

## Discussion

Our findings indicate that the A allele in rs4073259 is markedly increased in cerebral infarction patients in comparison to control subjects of Han ancestry in northern China. Genotype rs9579646 GG and rs9551963 AC were positively, while rs4073259 GG was negatively associated with cerebral infarction. Haplotypes rs9315050 & rs9551963 AAAC were positively associated with cerebral infarction. Furthermore, genotypes rs4147064 CT and rs9551963 AC were associated with cerebral infarction in patients with hypertension, while rs9579646 GG and rs4073259 GG were associated with cerebral infarction in patients with diabetes. The findings suggest that SNP rs4073259 of the *ALOX5AP* gene is associated with developing cerebral infarction in this cohort, although the possibility that it is a functional variant cannot be ruled out. Although we found no marked difference in the frequency of alleles of other *ALOX5AP* SNPs tested between the control and case groups studied, we found significant differences of genotype frequency of other *ALOX5AP* SNPs between the control and case subjects, suggesting that they might play a role in the pathogenesis of cerebral infarction in the Han population of northern China.

Microarray assays have revealed positive linkage domains associated with cardiovascular and cerebrovascular diseases which locate at 13p12-13 [1-3,10,14]. The ALOX5AP gene is located at bands 2 and 3 of region 1 of the long arm of chromosome 13, and encodes 5-lipoxygenase activating protein (FLAP) which regulates the synthesis of leukotrienes [15]. An increase in the production of leukotrienes and resulting inflammatory changes in local blood vessels may be associated with development of atherosclerosis [4,16-19]. Whether the rs4073259 SNP alters FLAP warrants further study.

The association between the genetic polymorphism of *ALOX5AP* and the occurrence of cerebral infarction has been previously reported, but discrepancies between studies carried out in different populations have been noted. For example, Kaushal *et al.*[9] found that rs957646 and rs769874 were significantly associated with stroke in whites in the US, but no association was found in blacks. Another US study by Zee *et al.*[6] found no association of HapA or Hap B with stroke. Meschia *et al.*[4] found no association of *ALOX5AP* variants and ischemic stroke in a US population, Zhang *et al.*[5] reported the *ALOX5AP* variant SG13S114T/A was associated with increased risk of stroke in Chinese males, and Linsel-Nitschke *et al.*[20] found HapB was associated with an increased risk of myocardial infarction in a German population. Ji *et al.*[21] in a recent study reported that the −581_582 Ins A polymorphism in *ALOX5AP* might be a genetic risk factor for ischemic stroke in the Chinese Han population. The lack of consistency in available studies also suggests a limit of the analytical methodologies used in these studies, the population association test. A better refined methodology, such as the Family base association test (FBAT), may be needed to provide consistent results [22].

Zintaras *et al.*[13] performed a meta-analysis in 2009 including all studies of *ALOX5AP* genotyping (5,194 stroke cases and 4,566 controls). The authors found significant heterogeneity among studies (PQ = 0.03, I2 = 63%), a non-significant association between the HapA and stroke risk (random-effects [RE] OR = 1.13, 95% CI 0.88-1.45), and no association of HapB with stroke risk (RE OR = 1.03, 95% CI 0.77-1.37). They also reported that the SG13S114, SG13S89, SG13S25, SG13S32, SG13S35, and SG13S42 polymorphisms were not associated with stroke. The authors concluded that to date, the cumulated evidence did not support an association of *ALOX5AP* variants and risk of stroke, though they cautioned that the conclusion was based on a relatively small number of studies.

The primary limitation of this study is the relatively low case number, especially in stratification based on genotypes. Further study with a larger population is required to confirm the findings. In addition, we did not study SNPs from other susceptible genes, *e.g.*, phosphodiesterase 4D (*PDE4D*) in which some of the SNPs have been shown to be associated with the development of cerebral infarction [4].

## Conclusions

In summary, our results indicate the *ALOX5AP* SNP A allele in rs4073259, genotype rs9579646 GG, rs9551963 AC, and haplotype rs9315050&rs9551963 AAAC were associated with an increased risk of ischemic stroke in the Han population, while rs4073259 GG was associated with a decreased risk. Genotype rs4147064 CT and rs9551963 AC were positively significantly associated with cerebral infarction in patients with hypertension while genotype rs9579646 GG was positively significantly associated with, while genotype rs4073259 GG was negatively significantly associated with, cerebral infarction in patients with diabetes. However, more studies with large sample size are needed to detect the extended haplotype, and further confirm whether other SNPs of the *ALOX5AP* gene are associated with cerebral infarction.

## Competing interests

The authors declare they have no competing interests.

## Authors’ contributions

We declare that all the listed authors have participated actively in the study, and all meet the requirements of the authorship. Dr. SZ designed the study and wrote the protocol. Dr. CZ performed research. Dr. MX contributed important reagents. Dr. CZ, Dr. ZQ, and Dr. ZZ managed the literature searches and analyses. Dr. SZ performed the statistical analysis. Dr. SZ wrote the first draft of the manuscript. BZ acquired blood samples. Dr LZ supervised the whole study. All authors read and approved the final manuscript.

## Funding

This study was funded by the Provincial Health Department of Heilongjiang Province, (project numbers 2006–207), the Provincial Science and Technology Department of Heilongjiang Province (project number GC08C411), and the Provincial Education Department of Heilongjiang Province (project number 11521139).

## Pre-publication history

The pre-publication history for this paper can be accessed here:

http://www.biomedcentral.com/1471-2350/13/61/prepub
